# Laparoscopic Cholecystectomy for Symptomatic Cholecystic Disease in Children: Defining Surgical Timing

**DOI:** 10.3389/fped.2020.00203

**Published:** 2020-05-08

**Authors:** Gloria Pelizzo, Rossana Bussani, Annalisa De Silvestri, Marco Di Mitri, Gregorio Rosone, Salvatore Amoroso, Mario Milazzo, Vincenza Girgenti, Giovanni Battista Mura, Elettra Unti, Davide Rozze, Vennus Shafiei, Valeria Calcaterra

**Affiliations:** ^1^Pediatric Surgery Unit, V. Buzzi Children's Hospital and Department of Biomedical and Clinical Science L. Sacco, University of Milano, Milan, Italy; ^2^Institute of Pathologic Anatomy, University of Trieste, Trieste, Italy; ^3^Biometry and Clinical Epidemiology, Scientific Direction, Fondazione IRCCS Policlinico San Matteo, Pavia, Italy; ^4^Pediatric Surgery Unit, Children's Hospital, ARNAS Civico-Di Cristina-Benfratelli, Palermo, Italy; ^5^Pathology Unit, ARNAS Civico-Di Cristina-Benfratelli, Palermo, Italy; ^6^Pediatric Unit, Department of Internal Medicine, University of Pavia and Fondazione IRCCS Policlinico San Matteo, Pavia, Italy

**Keywords:** laparoscopic, cholecystectomy, symptomatic, cholecystic disease, children, surgical timing

## Abstract

**Background:** Laparoscopic cholecystectomy (LC) is the standard of care for gallbladder (GB) pathologies. We evaluated clinical, ultrasonographic (US) data as well as histopathological findings in children affected with symptomatic cholecystic disease (SCD) who underwent LC, with the aim of defining surgical timing.

**Methods:** We reviewed our cases who underwent elective LC (ELC) or urgent LC (ULC). Clinical, US, surgical and histological features were used to create different risk scores.

**Results:** We considered 26 children (17 ELC/9 ULC). US signs were not different in the two groups (*p* > 0.05). Operating times were longer in ELC than in ULC (*p* = 0.01). Histopathological evaluation revealed fibrosis and atrophy in both ELC and ULC. The clinical risk score was higher in ELC compared to ULC (*p* < 0.001). An increased operative risk score was noted in patients with systemic inflammatory signs (OR1.98), lithotherapy (OR1.4.3) and wall thickening ≥3 mm (OR2.6). An increased histopathological risk score was detected in children with symptom duration >7 days (OR3.61), concomitant hematological disease (OR1.23) and lithotherapy (OR3.61).

**Conclusion:** Criteria adopted in adults cannot be adopted to detect the severity of GB damage in children. A dedicated clinical and US score is mandatory to define the most appropriate surgical timing.

## Introduction

The spectrum of gallbladder (GB) disorders in the pediatric population has changed over the past three decades (1). While they were previously largely attributable to hemolytic diseases, the prevalence of pediatric cholelithiasis has proportionally increased with the childhood obesity epidemic (2, 3).

Additionally, the improved survival of critically ill newborns who have received long-term parenteral nutrition or have underlying abnormalities resulting in short-bowel syndrome is considered a new factor related to the increased incidence of cholelithiasis in children (1, 4, 5). In turn, increases in the incidence of cholecystectomies have been described in pediatrics (1–3).

Laparoscopic cholecystectomy (LC) for calculous cholecystitis is the current standard of care for GB pathologies in children (6). To date no evidence regarding a correlation between clinical severity and histopathological findings have been identified in the pediatric age. While preoperative scoring systems have been created for adults, (7, 8). specific criteria supporting surgical timing have not been approved in children.

In this study, we retrospectively reviewed the clinical and sonographic data and histopathological findings in patients affected with symptomatic cholecystic disease (SCD), who underwent LC.

Data were analyzed to support the best indications for surgical timing in the pediatric age.

## Patients and Methods

### Patients

Data were retrospectively analyzed from December 2016 to March 2019 in patients who were admitted to our Surgical Department with a diagnosis of abdominal pain and suspicion of symptomatic cholecystic disease and subsequently underwent LC.

Clinical characteristics, sonographic features, surgical procedures, outcomes, and histopathologic evaluation were recorded in all patients. According to their clinical presentation, ultrasound parameters and inflammatory status, the enrolled patients were divided into two groups:

(1) Group 1, Elective Laparoscopic Cholecystectomy (ELC). This group included children presenting with painful abdominal symptoms for more than seven days and within 6 months of the earliest symptom referred by the patient: simple cholelithiasis, local Murphy's sign, right upper quadrant mass/pain/tenderness at first examination, without signs of inflammation and previous lithotherapy.

Conservative management included 3 months medical treatment with ursodeoxycholic acid (dose 10–20 mg/kg/day), clinical examination and US evaluation approximately once a month.

(2) Group 2, Urgent Laparoscopic Cholecystectomy group (ULC). This group included children who presented with right upper quadrant mass/pain/tenderness, local Murphy's sign and systemic signs of fever, elevated C-reactive protein, elevated white blood cell count, complicated cholelithiasis (cholecystitis or choledocholithiasis). Absence of symptom resolution and biochemical changes in inflammation (according to the 2018 Tokyo Guidelines) were considered indications for ULC within the first 7 days of symptom onset (9).

The study was approved by the Institutional Review Board of our center and was conducted in accordance with the 1975 Helsinki Declaration. The written consent for participation in the study is obtained.

### Methods

#### Data Collection

Data collection included the following variables: (1) clinical and biochemical data, (2) ultrasound features, (3) operative details, and (4) histopathological results.

#### Clinical and Biochemical Data

Clinical data included: age, gender, clinical features, previous abdominal surgeries, duration of symptoms, laboratory findings (including liver panel). Clinical features were used to create a clinical risk score, including: symptom duration >7 days, local Murphy's sign, systemic inflammatory signs, concomitant hematological disease, previous treatment with lithotherapy. For each parameter, a dichotomous variable was created (0 = absence and 1 = presence of these parameters, respectively).

#### Ultrasound Features

For all patients a sonographic investigation was performed with the ATL 5,000 ultrasound instrument using a 12–5 MHz linear array transducer in longitudinal and transverse planes, in the supine position.

The US study score included the presence of:

- specific signs including gallstones (single or multiple) and sonographic Murhpy sign (maximal tenderness from ultrasound probe pressure over the GB)
- aspecific signs including wall thickening ≥ 3 mm, gallstone dimensions >3 mm, GB distension, presence of pericholecystic fluid (10, 11).

A US score was defined using the parameters detailed in Table 1, which scaled the severity of the condition. For each parameter, a dichotomous variable was created (0 = absence and 1 = presence of these parameters, respectively).

**Table 1:** Sonographic signs of cholecystitis and score.

**Specific signs**	**Score**
**Gallstones**	
• Single	0
• Multiple	1
**Sonographic Murphy sign**	
(maximal tenderness with transducer pressure over the GB)	
• No	0
• Yes	1
**Aspecific signs**	
**Wall thickening**	
• <3 mm	0
• >3 mm	1
**Gallstone dimensions**	
• <3 mm	0
• >3 mm	1
**Gallbladder distention**	
• No	0
• Yes	1
**Pericholecystic fluid**	
• No	0
• Yes	1

#### Surgical Procedure and Data Recorded

The standard four-port surgical approach was utilized with: trans-umbilical open access, three trocars (2 operative and 1 accessory), cystic artery and ligation of the duct with non-absorbable polymer clips (knots or Hem-o-lok clip) and GB removal through the umbilical access.

All LC were performed by surgeons with more than 5 years' experience.

Operative details, including approach, duration and intraoperative events, post-operative complications, post-operative recovery were recorded.

As reported in Table 2, an operative grading system/score was adopted (12) to define surgical findings, which may also be useful in the pediatric laparoscopic cholecystectomy. Also here for each parameter, a dichotomous variable was constructed (0 = absence and 1 = presence of these parameters, respectively).

**Table 2:** Intraoperative macroscopic features.

**Intraoperative features**	**Score**
Gallbladder appearance	
• Adhesions <50%	0
• Adhesions GB >50%	1
Gallbladder	
• Distention/contraction	1
• Unable to grasp	1
• Stone impact	1
Severe complication	
• Signs of inflammation	1
• Time to identify cystic artery/duct > 90 min	1

#### Histological Analysis

The excised specimens, after fixation, were sectioned on multiple levels, processed and paraffin embedded. The blocks were cut into 2 μm axial sections. After hematoxylin/eosin staining, histological analysis was performed with an Olympus® Bx61 light microscope by a dedicated pathologist who evaluated all relevant features of the GB wall.

The histological parameters (with severity grading) used to define chronic cholecystitis are reported in Table 3. In order to identify the severity of the condition, a histopathological score was defined using these parameters.

**Table 3:** Histological parameters (with severity grading) used to define chronic cholecystitis and score.

**Parameters**	**Score**
**Ulcers and/or erosion**	
• No	0
• Yes (grade 1-2-3)	1
**Inflammatory cell infiltration**	
• No	0
• Yes	1
**Fibrosis**	
• No	0
• Yes (grade 1-2-3)	1
**Adenomyosis**	
• No	0
• Yes	1
**Reactive epithelial hyperplasia**	
• No	0
• Yes (grade 1-2-3)	1
**Epithelial atrophy**	
• No	0
• Yes (grade 1-2-3)	1
**Parietal atrophy**	
• No	0
• Yes (grade 1-2-3)	1
**Intramural microlitiasis**	
• No	0
• Yes (grade 1-2-3)	1
**Intestinal metaplasia**	
• No	0
• Yes	1

### Statistical Analysis

Qualitative variables were described as counts and percentages. Quantitative variables were expressed as the mean value and standard deviation (SD) when normally distributed (normal distribution was tested using the Shapiro-Wilk test) or median with interquartile range. Statistical analyses were performed using the χ^2^ test or the exact Fisher test for comparison of categorical variables and the Student's *t*-test or the Mann-Whitney U test for continuous variables. A *p*-value below 0.05 was considered statistically significant (tests two-sided). Association of variables and score was evaluated fitting ordinal logistic regression models.

STATA statistical package (release 15.1, 2017, Stata Corporation, College Station, Texas, USA) was used to perform the data analysis.

## Results

A total of 29 cholecystectomies were performed in our Unit during the enrollment period. Records of three children were missing or the pathological appearance did not meet study inclusion criteria, so they were excluded from the study.

### Patient Features

We considered 26 patients (14M/12F), with a mean age of 10.38–0.69 yrs (range age: 3 months−15 yrs). In seven cases, concomitant haematologic disease was recorded (6 hereditary spherocytosis, 1 thalassemia major). Seventeen (65.4%) patients were included in the ELC group and nine subjects (34.6%) in the ULC group. The features of the patients are reported in Table 4.

**Table 4:** Clinical and ultrasonographic features.

**Features**	**ELC group *N* = 17**	**ULC group *N* = 9**	***p***
**Gender**	9F/8M	3F/6M	0.3
**Age**	11.08–0.77	9.06–1.32	0.1
**Concomitant hematological disease**			
Yes	3/17 (17.65%)	4 (44.44%)	0.1
No	14/17 (82.5%)	5 (55.56%)	
**Splenomegaly**			
Yes	3/17 (17.65%)	4 (44.44%)	0.1
No	14/17 (82.5%)	5 (55.56%)	
**Hepatomegaly**			
Yes	0	2/9 (22.22%)	0.07
No	17/17 (100%)	7/9 (77.77%)	
**Previous abdominal surgeries**			
Yes	2/17 (11.76%)	4/9 (44.44%)	0.9
No	15/17 (88.24%)	5/9 (55.56%)	
**Pathological liver enzymes**	1/17 (5.88%)	3/9 (33.33%)	0.06
**Ultrasonographic parameters**			
• gallstones (no. of cases, %)			
- absent	1/17 (5.88%)	0	
- single	12/17 (70.59%)	6/9 (66.67%)	0.6
- multiple	4/17 (23.35%)	3/9 (33.33%)	
• sonographic Murhpy's sign (no. of cases, %)	8/17 (47.05%)	3/9 (33.33%)	0.6
• gallstone dimensions >3 mm (no. of cases, %)	8/17 (47.05%)	4/9 (44.44%)	0.8
	6/17 (35.29%)	3/9 (33.33%)	0.9
• gallbladder distension (no. of cases, %)	6/17 (35.29%)	6/9 (66.67%)	0.2
• wall thickening ≥ 3 mm (no. of cases, %)			

No significant differences between gender (*p* = 0.3) and age (*p* = 0.1) were noted between the groups.

Splenomegaly was found in 3/17 (17.6%) ELC and 4/9 (44.4%) ULC (*p* = 0.1) and hepatomegaly in 2/9 (22.2%) ULC (*p* = 0.1).

Biochemical parameters were similar in both groups, Table 4. No significant differences regarding previous abdominal surgeries were recorded between the groups (*p* = 0.9).

### Ultrasound Features

As reported in Table 4, the presence of specific US signs was similar in the two groups (gallstones *p* = 0.68, sonographic Murphy sign *p* = 0.6). Aspecific features (gallstone dimensions >3 mm, *p* = 0.8; gallbladder distension *p* = 0.9; wall thickening ≥ 3 mm, *p* = 0.2) were not different in ELC compared to ULC. No patients exhibited the presence of organ adhesions, pericholecystic fluid or peritoneal fluid.

In 2 children, one in the ECL group and one in the ULC group, anatomical abnormalities were detected (*p* = 0.6).

### Surgery

The mean operative time was significantly longer in ELC than in ULC (173.6–69.9 min vs. 112.7–53.7 min, *p* = 0.01).

Adhesiolysis was required in 10/17 (58.82%) ELC and in 6 (66.67%) ULC, *p* = 0.69. Hepatic tissue hemostasis was more frequently required in ULC than in ELC (*p* = 0.04). The identification of frailty (*p* = 0.1) and macroscopic signs of wall inflammation (*p* = 0.2), were similar in both groups.

Hilar lymphnode involvement was recorded in 7/17 ELC and 4/9 ULC (*p* = 0.87).

In seven cases (4 ECL and 3 ULC, *p* = 0.59), intraoperative cholangiography was necessary to clean the biliary tree. Intraoperative spillage was recorded in two cases in the ULC group (*p* = 0.04). Conversion to laparotomy and drainage positioning were not necessary in any subject.

In every patient, the GB was removed through the umbilical port without complications. No post-operative complications occurred in either group. No major morbidities, including recurrent pancreatitis, were observed nor any deaths in either group.

A longer post-operative stay was recorded in ULC compared to ELC (10.1 ± 5.0 and 5.1 ± 1.4 days *p* = 0.02).

### Histopathology

The histological parameters and their severity grading are reported in Table 5. Severe damage of the GB was detected in both groups. Regarding the two groups, there were no significant differences at the level of ulcers and/or ulcerations (*p* = 0.70), phlogosis (*p* = 0.06), inflammatory cell infiltration (*p* = 0.62), fibrosis (*p* = 0.37), adenomyosis (0.33), reactive epithelial hyperplasia (0.06), epithelial atrophy (*p* = 0.59); parietal atrophy (*p* = 0.22); intramural microlitiasis (*p* = 0.46); intestinal metaplasia (*p* = 0.28). In 2/3 patients with metaplasia, a multiseptate GB was detected.

**Table 5:** Histological features in the Elective Laparoscopic Cholecystectomy (ELC) and Urgent Laparoscopic Cholecystectomy (ULC) groups.

	**ELC group *n* (%)**	**ULC group *n* (%)**	***p***
**Ulcers and/or erosion**			
Grade 1	7 (41.18%)	4 (44.44%)	0.70
Grade 2	8 (47.06%)	3 (33.33%)	
Grade 3	2 (11.76%)	2 (22.22%)	
Inflammatory cell infiltration^*^			
Yes	14 (82.35%)	6 (66.67%)	0.34
No	3 (17.65%)	3 (33.33%)	
**Fibrosis**			
Grade 1	9 (52.94%)	4 (44.44%)	0.37
Grade 2	8 (47.06%)	4 (44.44%)	
Grade 3	0	1 (11.1%)	
**Adenomyosis**			
Yes	10 (58.82%)	1 (11.1%)	0.34
No	7 (41.18%)	2 (22.2%)	
**Reactive epithelial hyperplasia**			
Grade 0	4 (23.53%)	7 (77.78%)	0.06
Grade 1	5 (29.41)	1 (11.11%)	
Grade 2	6 (35.29%)	1 (11.11%)	
Grade 3	2 (11.76%)	0	
**Epithelial atrophy**			
Grade 0	3 (17.65%)	1 (11.11%)	0.59
Grade 1	5 (29.41%)	1 (11.11%)	
Grade 2	5 (29.41%)	3 (33.33%)	
Grade 3	4 (23.53%)	4 (44.44%)	
**Parietal atrophy**			
Grade 0	2 (11.76%)	2 (22.22%)	0.22
Grade 1	3 (17.65%)	3 (33.33%)	
Grade 2	6 (35.29%)	0	
Grade 3	6 (35.29%)	4 (44.44%)	
**Intramural microlitiasis**			
Grade 0	8 (47.06%)	5 (55.56%)	
Grade 1	7 (41.18%)	2 (22.22%)	0.46
Grade 2	2 (11.76%)	1 (11.11%)	
Grade 3	0	1 (11.11%)	
**Intestinal metaplasia**			
Yes	2 (11.76%)	1 (11.1%)	0.28
No	15 (88.24%)	0	

* *No differences in type of infiltrating cells were noted (p = 0.6)*.

### Risk Score

The clinical risk score was higher in ELC compared to ULC (*p* < 0.001). No significant differences were noted in US, operative and histopathological risk scores (*p* = 0.49, *p* = 0.36, *p* = 0.17, respectively).

An increased operative risk score was noted in patients with systemic inflammatory signs (OR 1.98, *p* = 0.35), previous treatment with lithotherapy (OR 14.3, *p* = 0.003) and US detection of wall thickening ≥ 3 mm (OR 2.6, *p* = 0.17).

An increased histopathological risk score was detected in children with a symptom duration >7 days (OR 3.61, *p* = 0.12), concomitant hematological disease (OR 1.23, *p* = 0.77) and prior treatment with lithotherapy (OR 3.61, *p* = 0.12).

## Discussion

Cholelithiasis is a relatively uncommon pediatric disease, with a prevalence between 0.13 and 0.22% that is currently on the rise (13). Around 80% of patients with gallstones remain asymptomatic (14, 15). Gallstones can block the cystic duct causing GB distension and biliary colic. Prolonged obstruction results in inflammation, infection, and even ischemia, resulting in acute inflammation of GB; repeated episodes can result in chronic cholecystitis, in which a thickened GB wall, GB mucosal atrophy and scarring were detected (16).

There is no consensus on the management of children with cholelithiasis and the risks of developing complications as a consequence of delaying surgery are well-known (17–19). More than 70% of cases admitted in emergency became symptomatic when surgery is delayed after a mean time of 38 months. Additionally, the overall incidence of cholecystitis in childhood is divided to include the calculous form and the acalculous form. The latter is reported to be increasing in the pediatric population (20, 21).

At the early stage, the diagnosis of acute GB diseases is also difficult because symptoms are non-specific and a clinical manifestation of the presence of cholecystitis, such as Murphy's sign, may be difficult to detect in children. Thus, abdominal ultrasonography (US) is considered the most accurate diagnostic technique, it is also cost-effective, free of radiation hazards and highly specific to the biliary system (22, 23). On the other hand, there is a well-known, significant intra- and inter-observer risk in US evaluation. Additionally, as reported by Tsai, ultrasound findings in pediatric cholecystitis have lower sensitivities and positive predictive values than those reported in adults (24, 25).

LC has gradually become the technique of choice for symptomatic gallstones also in the pediatric age (6). Compared with open cholecystectomy, this procedure has several advantages including less post-operative pain, a short recovery period, faster return to unrestricted activities and better cosmetic results. Indications for surgery include symptomatic cholelithiasis unresponsive to medical treatment, no longer recommended in the pediatric population (26). Several predisposing factors, including hemolytic disorders and obesity, may create problems of the GB that require surgery (26).

The severity of the GB pathological condition is a key factor in determining the feasibility of the laparoscopic approach and defining the surgical timing (26). Patients with difficult cholecystectomies are at significantly higher risk of biliary duct injury conversion rate, general complications and longer surgical times. Intense inflammation and firm adhesions of the GB with the common bile duct, duodenum or even the colon due to complicated cholecystitis make dissection and identification of the anatomy very difficult (27, 28). Bleeding during dissection further hinders the safe identification of anatomy. According to the Safe Cholecystectomy Program of the Society of American Gastrointestinal and Endoscopic Surgeons, achieving the Critical View of Safety is defined by three criteria: the hepato-cystic triangle is cleared of fat and fibrous tissue; the lower one third of the GB is separated from the liver to expose the cystic plate and no more than two structures should be seen entering the GB (28).

Our results showed that both ELC and ULC are safe and feasible in children. The clinical parameters and ultrasonographic criteria currently used in adults (7, 8) to define the timing of LC do not allow the severity prediction of the GB histopathological condition and consequently the surgical outcome in children. We observed severe damage of the GB in both groups, including chronic signs of inflammation and pre-cancerous conditions, such as metaplasia. These features suggest that children may have milder episodes of self-limited GB inflammation compared with adults, which may lead to delayed treatment (24).

Traditionally, GBs removed for presumed benign disease are sent for histopathological examination, but this practice has been the subject of controversy. Considering the very low incidence of pre-malignant and malignant lesions of the gallbladder, some authors recommend selective histopathological examination of cholecystectomy specimens with abnormal macroscopic findings (29–32).

We suggest routine histopathological examination for all cholecystectomy specimens removed for benign GB diseases in the pediatric age, in order to detect metaplasia. As reported by the Royal College of Pathologists (33) oncological diagnosis can be missed, particularly in patients with a multiseptate GB. This condition is considered an indication for surgery and a risk factor for developing GB degeneration.

While a number of preoperative scoring systems are utilized in adults, (7, 8) in pediatric patients there is no useful classification to predict prognosis and outcome. The results of the present study, revealed that the identification of dedicated preoperative clinical-ultrasound scores for the pediatric age that are predictive of disease severity should allow optimization of surgical timing. According to our risk score, long duration of symptoms, systemic inflammatory signs, previous lithotherapy and wall thickening ≥3 mm should be considered as crucial parameters to detect severe forms that necessitate immediate surgery, as in adults. On the contrary, some parameters, such as age, sex, history of abdominal surgery, which are usually used in adult scoring systems are not useful in pediatrics. Finally ultrasound parameters such as hydropic gallbladder (diameter > 4.5 cm) and shrunken gallbladder (8) are not adequate in pediatrics due to the differences in the measurements for a normal GB, which are related to height, weight, and body surface area, during the development age (34).

We recognize that there are some limitations in this study starting from the limited sample size. Therefore, a larger cohort of patients is mandatory to confirm these results. Additionally, the lack of clinical and auxological data (i.e., BMI, neonatal history) may have limited the interpretation of symptoms and US features and these could represent possible factors influencing intraoperative difficulty.

In conclusion, the criteria defining the timing of surgery are not available, nor is there consensus on criteria grading the severity of GB damage in pediatric population. Clinical and US scores are mandatory to define appropriate surgical timing in children.

## Data Availability Statement

The datasets generated for this study are available on request to the corresponding author.

## Ethics Statement

The studies involving human participants were reviewed and approved by ARNAS Civico-Di Cristina-Benfratelli. Written informed consent to participate in this study was provided by the participants' legal guardian/next of kin.

## Author Contributions

GP designed experiments, performed surgical interventions, wrote, and supervised the paper. VC designed experiments, wrote, and supervised the paper. MD recorded data of the patients. GR, SA, MM, VG, and GM performed surgical interventions. AD performed statistical analysis. RB performed histological evaluation, wrote, and supervised the manuscript. EU, DR, and VS performed histological evaluation. All authors have read and approve of the manuscript.

## Conflict of Interest

The authors declare that the research was conducted in the absence of any commercial or financial relationships that could be construed as a potential conflict of interest.
